# Multicenter Evaluation of a Novel Immunochromatographic Test for Anti-aspergillus IgG Detection

**DOI:** 10.3389/fcimb.2019.00012

**Published:** 2019-01-31

**Authors:** Raphaël P. Piarroux, Thomas Romain, Aurélie Martin, Damien Vainqueur, Joana Vitte, Laurence Lachaud, Jean-Pierre Gangneux, Frédéric Gabriel, Judith Fillaux, Stéphane Ranque

**Affiliations:** ^1^LDBIO Diagnostics, Lyon, France; ^2^Aix-Marseille Université, APHM, IRD, SSA, VITROME, IHU-Méditerranée Infection, Marseille, France; ^3^APHM, Parasitologie-Mycologie, IHU Méditerranée Infection, Aix-Marseille Université, Marseille, France; ^4^Service de Parasitologie–Mycologie, CHU de Toulouse, Toulouse, France; ^5^APHM, MEPHI, IHU Méditerranée Infection, Aix-Marseille Université, Marseille, France; ^6^CHU de Montpellier, Montpellier, France; ^7^Université de Montpellier, Montpellier, France; ^8^Institut de Recherche en Santé, Environnement et travail, Inserm, CHU de Rennes, EHESP, Université de Rennes, UMR_S 1085, Rennes, France; ^9^Laboratoire de Parasitologie-Mycologie, CHU Bordeaux, Bordeaux, France; ^10^PHARMA-DEV, IRD UMR 152, Université Paul Sabatier, Toulouse, France

**Keywords:** immunochromatography, *Aspergillus* serology, point-of-care, chronic pulmonary aspergillosis, allergic broncho-pulmonary aspergillosis, sensitivity, specificity

## Abstract

*Aspergillus* sp. fungi cause various diseases in both immunocompetent and immunocompromised patients. The most frequent *Aspergillus* disorders include chronic pulmonary aspergillosis (CPA), a life-threatening disease that affects at least 3 million people worldwide, and allergic bronchopulmonary aspergillosis (ABPA), which affects approximately 4.8 million severe asthmatic patients globally. Diagnosis of such diseases involves IgG serological testing; however, the currently available anti-*Aspergillus* IgG detection assays are inappropriate for resource-poor laboratory settings, as they are expensive, rely on automated procedures, and require stable electrical power. Therefore, accurate CPA or ABPA diagnosis facilities are lacking in most low- and middle-income countries. We evaluated a novel anti-*Aspergillus* antibody immunochromatographic test (ICT) that requires minimal laboratory equipment. Two evaluations were performed: a single-center 4-month prospective study in a French reference laboratory (44 cases/257 patients) and a retrospective study in five French reference laboratories (262 cases and 188 controls). We estimated the ICT indices for the diagnosis of chronic aspergillosis, and the test results were compared to those of anti-*Aspergillus* IgG immunoblot (IB) assay. Of the 713 patients included in the study, 306 had chronic aspergillosis. Test sensitivity and specificity were 88.9% (95%CI[85–92]) and 96.3% (95%CI[94–98]) for the ICT and 93.1% (95%CI[90–96]) and 94.3% (95%CI[92–96]) for the IB, respectively. Agreement between the two assays was almost perfect (kappa = 0.86). As this ICT displays good diagnostic performance and complies with the ASSURED (Affordable, Sensitive, Specific, User-friendly, Equipment-free, and Delivered) criteria, we concluded that this anti-*Aspergillus* antibody ICT can be used to diagnose *Aspergillus* diseases in resource-poor settings.

## Introduction

*Aspergillus fumigatus* (*Af*) is a ubiquitous mold that spreads via airborne spores. Inhaled spores are usually cleared from the airway by the mucociliary transport and innate immune systems. However, in certain frail patients, the fungus can cause various diseases, which are classified into six major groups: transient or chronic colonization, immune-allergic sensitization, chronic pulmonary aspergillosis (CPA), allergic bronchopulmonary aspergillosis (ABPA), non-pulmonary localized aspergillosis, invasive aspergillosis (IA), and sub-acute invasive aspergillosis (SAIA) (Barnes and Marr, 2006).

Most forms of aspergillosis are observed in immunocompetent patients with chronic lung diseases, with the exception of IA and SAIA, which only occur in moderately or severely immunocompromised patients. CPA usually occurs in patients with pre-existing cavitary lung lesions, such as tuberculosis sequels. ABPA primarily occurs in asthmatic or cystic fibrosis patients. The worldwide prevalence of aspergillosis is approximately 8 million, predominantly including cases of ABPA (4.8 million) (Maturu and Agarwal, 2015; Carsin et al., 2017) and CPA (3 million, including 1.6 million post-tuberculosis cases; (Denning et al., 2011; Page et al., 2016). Although most patients are diagnosed in high-income countries, it has been estimated that the prevalence is higher in middle- to low-income countries (Denning et al., 2011). In France, most cases are immunoallergic forms, with approximately 95,000 cases of ABPA (Gangneux et al., 2016).

*Aspergillus*-related disease symptoms are heterogeneous and may be confused with many other pulmonary conditions. Therefore, the diagnosis of *Aspergillus*-related diseases is complex and relies on the combination of positive serology results with compatible clinical symptoms and radiological findings (Denning et al., 2016). To detect anti-*Aspergillus* antibodies, various serological techniques are currently available, ranging from precipitin-based techniques, such as immunoelectrophoresis (IEP), to automated ELISA or immunoblot (IB). However, assay performance varies greatly and discrepancies are common between the various techniques, as recently demonstrated by Page et al. (2016). This heterogeneity may be explained by the variability of antigens (either culture extract or recombinants) and/or type of assay reaction (ELISA, IEP, or IB). While other techniques detect only one IgG isotype, precipitin-based techniques are also capable of detecting both IgA and IgM. This distinction might explain why some patient results yield a positive precipitin test and a negative IgG test. Although, precipitin-based assays have a relatively lower sensitivity compared with other assays (Baxter et al., 2013). Furthermore, as such techniques are either expensive, require a stable electrical source, or involve complex automated systems, none of the currently available techniques are suited for resource-poor settings. Therefore, the capacity to properly diagnose *Aspergillus*-related diseases is significantly limited in the countries where it is most needed (Denning et al., 2011). Immunochromatographic tests (ICT) are easy to both use and read (usually via the naked eye). As such assays require minimal equipment, are less expensive than typical serological assays, and can be performed in small series, they are highly suited for resource-poor settings. Such assay has been developed for the detection of *Aspergillus* antigen (Thornton, 2008; Hoenigl et al., 2018), however, to our knowledge, no ICT assays are currently available for the detection of antibodies specific to *Aspergillus*, despite repeated demands (Page et al., 2015; Richardson and Page, 2017).

This study aimed to evaluate anti-*Aspergillus* antibody detection in serum samples using a novel ICT assay. This test uses colored latex particles that enable the detection of anti-*Aspergillus* IgG via the naked eye. The diagnostic performance of the ICT was evaluated on patient samples corresponding to various *Aspergillus*-associated diseases and then compared with the IB results.

## Materials and Methods

### Inclusion Criteria

Two studies were performed: a single- prospective study and a retrospective multicenter study. In both studies, only patients of at least 18 years of age were included. Patient medical history files, including routine serological test results, were first screened by a medical mycology expert. If a conclusion could not be reached by the expert, a decision was then made by consensus with a second expert. The prospective study was conducted at the University Hospital la Timone in Marseille, France. All sera received for *Aspergillus* serology from July 2017 to late-October 2017 were also tested with the new ICT in parallel with the routine techniques. All samples of sufficient volume were retrospectively tested via IB assay; a total of seven samples lacked ample volume for IB as further detailed. The retrospective study was conducted in five French university hospitals, located in Bordeaux, Marseille, Montpellier, Rennes and Toulouse. All centers included samples from patients with *Aspergillus* disease. Bordeaux, Marseille and Toulouse also included control sera, which were derived from patients for whom the diagnosis of *Aspergillus* disease had been excluded. All tested sera were collected between 2015 and 2018. To carry out the ICT and IB assays, including potential duplicates, a sample volume of 200 μL was required. Both IB and ICT assays were performed and interpreted blindly and anonymously.

### Case Definition

The following *Aspergillus* diseases were considered:

- Colonization was defined by two *Aspergillus* sp.-positive cultures from respiratory samples collected between ≥10 days apart and ≤6 months apart in a patient who did not meet other *Aspergillus*-related disease criterion.
- ABPA was defined according to Agarwal et al. (2013) as follows: elevated total IgE levels and elevated (>0.1 kU/l) *Af*-specific serum-IgE levels using the ImmunoCap® assay. The patient had either asthma or cystic fibrosis and at least one of the following elements: positive anti-*Aspergillus* IgG, *Aspergillus* colonization or compatible imaging.
- CPA was defined according to Denning et al. (2016) as follows: lung fungus ball and/or irregular intraluminal material and/or fibrotic destruction in one or more pulmonary lobe as shown via radiology and positive serology, culture-positive broncho-alveolar lavage or positive histological examination.
- IA was defined according to the proven/probable EORTC/MSG criteria (De Pauw et al., 2008). For the diagnosis of SAIA, underlying clinical conditions were extended to include less severe immunosuppression, such as intensive care unit admission, and evolution of symptoms over 1 month.
- Other proven localized aspergillosis, such as histologically confirmed *Aspergillus* abscesses or fungal sinusitis, were also considered. For fungal sinusitis, invasive, non-invasive and allergic forms were included according to Chakrabarti and Kaur (2016).

Control definitions:

Prospective study: patients who did not correspond to any of the case definitions above were considered non-*Aspergillus* disease patients.

Retrospective study: negative controls were selected as follows:

- In Bordeaux, sera collected from patients who underwent *Aspergillus* serology assessment but did not correspond to any of the case definitions above were used as negative controls.
- In Toulouse, sera collected from patients who had been screened before solid organ transplantation and displayed no marker of *Af* disease.
- In Marseille, sera derived from patients with other pulmonary infectious diseases, rheumatoid factor or anti-nuclear antibodies were selected to assess potential ICT cross-reaction.

### Serological Techniques

#### Routine Techniques

All commercialized techniques were used and interpreted according to manufacturer's instructions. Each center used its own routine laboratory assays to detect anti-*Aspergillus* IgG antibodies:

- Marseille: IEP (in-house, threshold: one precipitin band) and ELISA Serion® (Würzburg, Germany).
- Montpellier: ELISA Bio-Rad® (Marnes-la-Coquette, France), followed by confirmation of positive and equivocal results via IB using Aspergillus WB IgG (LDBIO Diagnostics, Lyon, France).
- Bordeaux: ELISA Bio-Rad and IB LDBIO.
- Toulouse: ELISA Serion and IEP (in-house, somatic and metabolic *Af* antigens, threshold: three bands for at least one of the two antigens).
- Rennes: ELISA Bio-Rad and IEP (in-house, threshold: two bands or catalase activity).

#### Immunochromatographic Test

The ICTs were provided by LDBIO. The composition of the test is detailed in the instructions provided with the kit and available online^1^.

**Figure 1:**
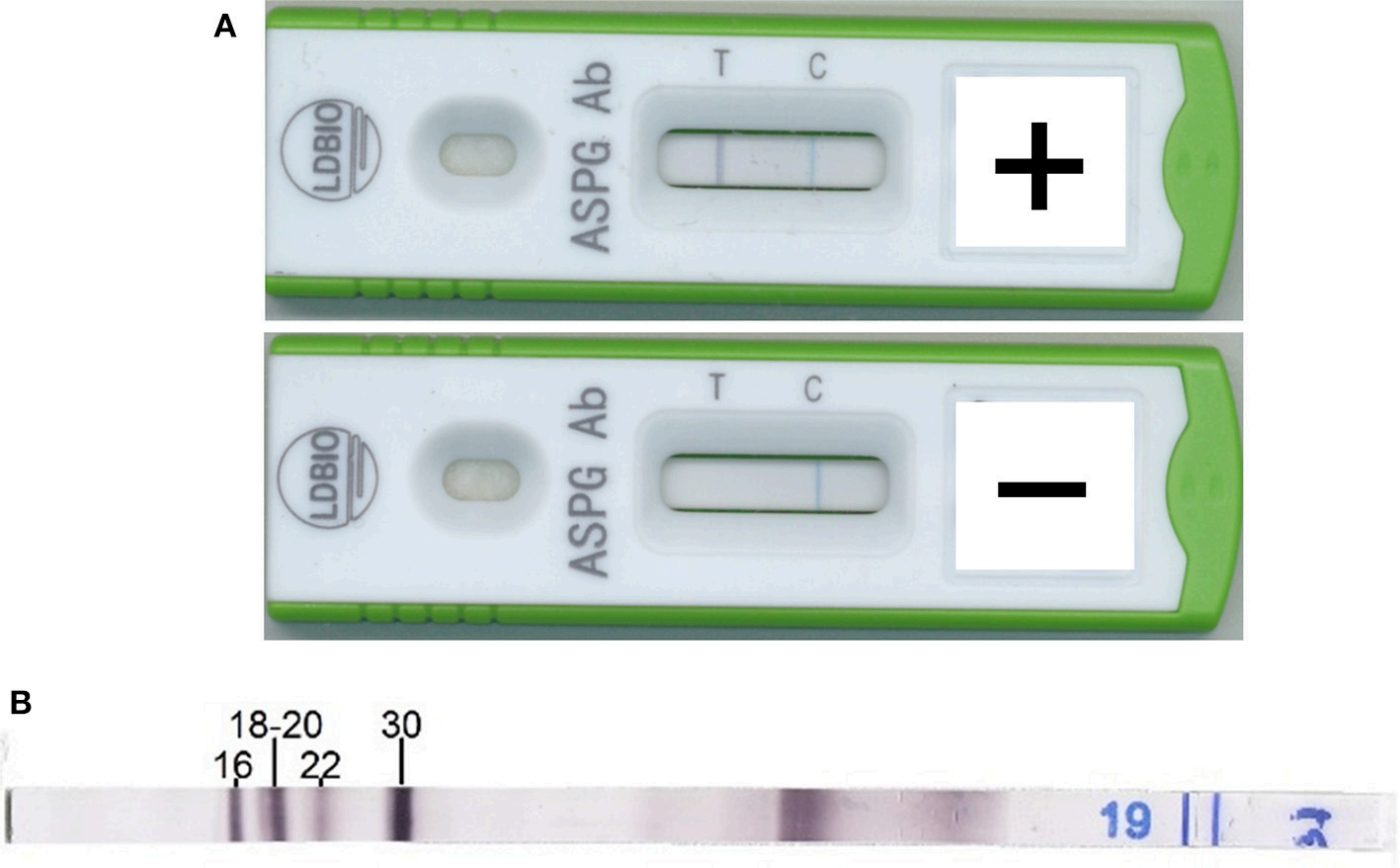
**(A)** Example of positive (top) and negative (bottom) immunochromatographic test results. **(B)** Positive Aspergillus Immunoblot results. Immunoblot results were considered positive if at least two bands between B16, B18-20, B22, and B30 were visible (by the naked eye). Band numbers correspond to the approximate molecular weight of the antigens in kDa.

The ICT was performed as follows: 15 μL of sample (serum or plasma) followed by four drops of the elution solution (provided with the kit) were aliquoted into the cassette sample well. The results were read between 20 and 30 min after adding the elution solution. The result was considered positive if a gray or black line was visible under the “T” marker; otherwise, the sample was considered negative. The test result was considered invalid if the blue “C” line did not appear. Examples of positive and negative ICT results are illustrated in Figure 1.

#### Immunoblot

All IBs were performed and interpreted according to the manufacturer's instruction. An example of a positive IB result is provided in Figure 1. When IB assays were performed during routine laboratory workup (Montpellier, Bordeaux), the results were collected from the hospital Laboratory Information Management System. The IBs were performed on site in Toulouse. The IBs were performed at LDBIO for Marseille and Rennes.

### Statistical Analysis

The sensitivity and specificity of the ICT and IB were calculated for the prospective and retrospective study populations as well as the overall population. For the prospective study, positive and negative predictive values were calculated for the ICT and IB. We calculated the 95% confidence intervals (95%CI) using binomial law (Clopper and Pearson, 1934). For both tests, the Diagnostic Odds Ratio (DOR) and corresponding 95%CI (Glas et al., 2003), Number needed to diagnose and misdiagnose (Habibzadeh and Yadollahie, 2013) were calculated for the overall population.

The strength of agreement between the ICT and IB was measured using the Cohen's kappa (Cohen, 1960) with the following scale: 0–0.2, very weak agreement; 0.21–0.4, weak agreement; 0.41–0.6, moderate agreement; 0.61–0.8, strong agreement; and 0.81–1, almost perfect agreement (Landis and Koch, 1977).

All other comparisons were performed using the chi-squared test.

### Ethical Considerations

*Aspergillus* serology was performed during routine laboratory work-up for the patients who received written laboratory reports. The IB and ICT assays were performed using excess serum. Patient characteristics were obtained from a non-interventional review of medical charts and laboratory results. According to French law, the patients were informed and retained the right to oppose the use of their anonymized medical data for research purposes. Dedicated ethical approval and individual patient consent were not necessary for this type of study^2^, ^3^ Patient data were anonymized as required by the French regulatory authorities (CNIL authorization n°2151008).

## Results

### Patient Recruitment

In the prospective study, *Aspergillus* serology was performed for 276 patients in Marseille during the 4-month period; 13 patients were excluded because they were <18 years of age. Of the 263 remaining samples, 44 were classified has having *Aspergillus* disease, while 219 samples were classified as controls. Patients distribution is detailed in Table 1. Due to limited sample volume, seven samples could not be tested by IB (two colonization and five negative samples).

**Table 1:** Distribution of the various clinical forms of Aspergillus disease and study population per center.

	**Prospective study**	**Retrospective study**				
	**Marseille**	**Bordeaux**	**Marseille**	**Montpellier**	**Rennes**	**Toulouse**
Colonization	24 (IB^*^: 22)$	20	6	13	1	31
ABPA^*^	6	27	8	18	4	14
CPA^*^	11	16	0	6	34	12
IA SAIA^*^	3	3	0	7	2	6
Others^*^	0	1	0	2	33	1
Total positive tests	44 (IB: 42)$	64	14	46	74	64
Negative controls	219 (IB: 214)$	68	43¤	0	0	77

* ABPA, allergic bronchopulmonary aspergillosis; CPA, chronic pulmonary aspergillosis; IA SAIA, invasive or sub-acute invasive aspergillosis; IB, Immunoblot; ICT, Immunochromatographic test; others: severe asthma with fungal sensitization, abscesses, Aspergillus sinusitis. ¤14 of the 43 samples were selected to assess potential test cross-reaction with other documented lung infections (mostly due to Streptococcus pneumoniae), 24 samples were selected to assess potential cross-reaction with rheumatoid factor, and five samples were selected to assess potential cross-reaction with anti-nuclear antibodies.

*$Seven samples were not done with IB due to limited volume: two colonization and five controls*.

Retrospective study: 262 sera from patients with aspergillosis and 188 controls were selected. Study populations and *Aspergillus* disease distribution per center are detailed in Table 1.

### ICT Results

The blue “C” line was visible for all cassettes; thus, all tests were interpretable. In the prospective study population, the ICT was positive in 40 of 44 cases, with 90.9% (95%CI [78.3–97.5%]) sensitivity, while the ICT was negative in 211 of 219 controls, with 96.3% (95%CI [92.9-98.4%]) specificity. The positive predictive value was 83.3% (95%CI [69.8–92.5%]), and the negative predictive value was 98.1% (95%CI [95.3–99.5%]).

In the retrospective study, the ICT showed 88.5% sensitivity (95%CI [84.1–92.1%]) and 96.3% (95%CI [92.5–98.5%]) specificity. Notably, of the 43 sera tested for potential cross-reaction, only one rheumatoid factor-positive serum yielded a positive ICT result. No cross-reaction was detected in the sera of patients infected with other etiological agents of pneumonia nor in those with anti-nuclear antibodies.

The differences between the diagnostic indices measured in the prospective and retrospective study were not statistically significant (*p* = 0.64).

In the global study population, the ICT showed 88.9% sensitivity (95%CI: [84.8–92.2%]), 96.3% specificity (95%CI [94.0–97.9%]), and a high DOR of 209 (95%CI: [112–391]) (Table 2). The number needed to diagnose and to misdiagnose were 1.04 and 14.5, respectively.

**Table 2:** The ICT and IB results of the prospective, retrospective and pooled studies.

	**Prospective study**		**Retrospective study**		**Overall**	
	**ICT**	**IB**	**ICT**	**IB**	**ICT**	**IB**
Cases (pos./neg.)	40/4	36/6	232/30	247/15	272/34	283/21
Controls (pos./neg.)	8/211	22/192	7/181	1/187	15/392	23/379
Sensitivity	90.9%	85.7%	88.5%	94.3%	88.9%	93.1%
Specificity	96.3%	89.7%	96.3%	99.5%	96.3%	94.3%

*Test results are presented as follows: XX/YY where XX is the number of positives tests and YY the number of negatives tests in the population. IB, Immunoblot; ICT, Immunochromatographic test*.

### IB Results

In the prospective study population, the IB showed good sensitivity (36/42, 85.7%, 95%CI [71.5–94.6%]) and specificity (192/214, 89.7%, 95%CI [84.9–93.4%]). The positive predictive value was 62.1% (95%CI [48.4–74.5%]), and the negative predictive value was 97.0% (95%CI [93.5–98.9%]).

Retrospective study: the IB showed 94.3% sensitivity (_95_CI [90.7–96.7%]) and 99.5% specificity (_95_CI [97.1–100%]).

In the global study population, the IB assay had 93.1% sensitivity (_95_CI: [89.6–95.7%]), 94.3% specificity (_95_CI [91.5–96.3%]), and a high DOR of 222 (95%CI: [121–409]) (Table 2). The number needed to diagnose and to misdiagnose were 1.1 and 16, respectively.

### Comparison of Diagnostic Indices Between Different Forms of *Aspergillus* Disease

The comparison of diagnostic indices between the different forms of *Aspergillus* disease was performed on the global study population. The results are summarized in Table 3. Briefly, the ICT had a lower sensitivity for the diagnosis of IA-SAIA than for ABPA (*p* = 0.0017), CPA (*p* = 0.0028) or colonization (*p* = 0.017). No other statistically significant difference was identified. Similarly, the IB assay showed a lower sensitivity for the diagnosis of IA-SAIA than for ABPA (*p* < 0.001) or CPA (*p* < 0.001), the IB assay also showed lower sensitivity for the diagnosis of colonization than for ABPA (*p* = 0.006) or CPA (*p* = 0.005).

**Table 3:** Comparison between diseases: results of ICT and IB for each Aspergillus-related disease.

***Aspergillus*-related disease**	**ICT**	**IB**
ABPA	69/74 (93%)	73/74 (99%)
CPA	73/79 (92%)	78/79 (99%)
Colonization	84/95 (88%)	82/93 (88%)
IA-SAIA	14/21 (67%)	16/21 (76%)
Others	32/37 (86%)	34/37 (92%)

*The results are displayed as the number of positive/negative results, via ICT and IB, for each group of Aspergillus-related diseases and the corresponding percentage. IB, Immunoblot; ICT, Immunochromatographic test*.

### Comparison of the ICT and IB

Test sensitivity did not significantly differ between the ICT and IB in the prospective and overall study populations nor for the diagnosis of each distinct *Aspergillus* disease. The specificity of the ICT was significantly higher in the prospective study populations (*p* = 0.007) but not in the global study population (*p* = 0.17).

The ICT and IB results were consistent in 657 of 706 samples (93%); the kappa coefficient was 0.858 (95%CI [0.819–0.896]), indicating an almost perfect agreement.

## Discussion

This novel anti-*Aspergillus* IgG ICT showed very good sensitivity (88.9%) and specificity (96.3%) for the diagnosis of *Aspergillus* diseases. The sensitivity was however lower for the diagnosis of acute and sub-acute forms of *Aspergillus* disease than for the other forms. One explanation might be that patients presenting with acute and sub-acute forms usually display a greater degree of immunosuppression than patients presenting with the other clinical forms, and thus might have lower serum antibody levels (Denning et al., 2016).

In our study, the ICT and IB results were comparable to those of others studies, using various techniques for anti-*Aspergillus* IgG detection, as shown in Table 4. Notably, the cut-off used for ELISA-based techniques are heterogeneous among studies. This difference is due to a high variation in antibody levels in the control group. For instance, a 20 mg/l cut-off yielded 98% specificity for Ugandan blood donors, whereas a 50 mg/l cut-off was required for European controls (Page et al., 2016, 2018). ICT specificity should also be assessed in other countries and populations, as our study was only carried out in France on a hospital-based population of patients who are at risk of aspergillosis.

**Table 4:** Comparison of overall test performance of the various anti-Aspergillus antibody detection techniques.

**Study**	**Technique/cut-off**	**Cases**	**Controls**
This study	ICT/any band	*Aspergillus* diseases, *n =* 30688.9%	Ruled out, *n =* 407 96.3%
	IB ≥2 bands	*Aspergillus* diseases, *n =* 30493.1%	Ruled out, *n =* 402 94.3%
Oliva et al., 2015	IB ≥2 bands	*Aspergillus* diseases, *n =* 308^*^91.2%	Blood donors, *n =* 212 93.9%
Page et al., 2016	ImmunoCap® ≥20 mg/l	CPA, *n =* 24196%	Blood donors, *n =* 100 98%
	Immulite® ≥10 mg/l	CPA, *n =* 24196%	Blood donors, *n =* 100 98%
	Serion® ≥35 U/ml	CPA, *n =* 24190%	Blood donors, *n =* 100 98%
	Dynamiker® ≥65 U/ml	CPA, *n =* 24177%	Blood donors, *n =* 100 97%
	Genesis® ≥20 U/ml	CPA, *n =* 24175%	Blood donors, *n =* 100 99%
	IPD/1 band	CPA, *n =* 24159%	Blood donors, *n =* 100 100%
Dumollard et al., 2016	Bordier® OD ≥0.8	CPA/ABPA, *n =* 22697%	Ruled out, *n =* 206 90.3%
	Bio-Rad® ≥5 U/ml	CPA/ABPA, *n =* 22691.7%	Ruled out, *n =* 206 91.3%
	Serion® ≥50U/ml	CPA/ABPA, *n =* 22686.1%	Ruled out, *n =* 206 81.5%
Guitard et al., 2012	Bio-Rad® ≥10 U/ml	CPA/ABPA, *n =* 6490.6%	Ruled out and pregnant, *n =* 371 89.6%
	Serion® ≥70 U/ml	CPA/ABPA, *n =* 6485.9%	Ruled out and pregnant, *n =* 371 84.4%

* Various forms of Aspergillus disease tested: ABPA, CPA, and colonization. Blood donors: blood donors considered negative for Aspergillus-related disease. Pregnant: Pregnant women without pulmonary condition and considered free of Aspergillus-related disease. Ruled-out: patients for whom diagnosis of aspergillosis had been ruled out.

*ABPA, allergic bronchopulmonary aspergillosis; CPA, chronic pulmonary aspergillosis; IB, Immunoblot; ICT, Immunochromatographic test IPD, immunoprecipitin detection*.

Finally, the ICT complies with the ASSURED criteria (Drain et al., 2014) in that the assay requires minimal laboratory equipment (serological pipette, clinical gloves and a centrifuge to separate serum from blood cells), is easy to use (interpretation via the naked eye), has good sensitivity and specificity, and operates under tropical or desert conditions (tested under 100 and 30% relative humidity, 37°C; data not shown). The assay can be stored at ambient temperature for at least 2 months after initial storage at 2–8°C before use and can therefore be used in environments with no reliable power source. The test could be used in resource-poor settings in complement to CT-scan (when available) or chest X-ray, when CT-Scan is not available, as recently proposed by Denning et al. (2018).

## Author Contributions

RP and SR designed the experiment. RP, TR, AM, and DV conducted the test. TR, AM, and SR reviewed medical files—prospective study. DV, JV, LL, FG, JF, J-PG, and SR reviewed medical files—retrospective study. RP and SR wrote the manuscript. All authors approved final version.

### Conflict of Interest Statement

RP is a Ph.D. student currently employed at LDBIO. SR received travel grants in relation to this work. The remaining authors declare that the research was conducted in the absence of any commercial or financial relationships that could be construed as a potential conflict of interest.

## Footnotes

^1^Ldbiodiagnostics. Aspergillus ICT IgG-IgM Instruction For Use [Internet]. (2018). Available at: http://ldbiodiagnostics.com/m-63-notices-d-utilisation.html [accessed Oct. 10th 2018]

^2^Loi n° 2012-300 du 5 mars 2012. Journal Officiel de la République Française (2012).

^3^Décret n° 2016-1537 du 16 novembre 2016. Journal Officiel de la République Française (2016).

## Abbreviations

ABPA: Allergic broncho-pulmonary aspergillosis
CPA: Chronic pulmonary aspergillosis
IA: Invasive Aspergillosis
IB: Immunoblot
ICT: Immunochromatographic test
IEP: Immunoelectrophoresis
SAIA: Sub-acute invasive aspergillosis.
